# Effect of Citrus Byproducts on Survival of O157:H7 and Non-O157 *Escherichia coli* Serogroups within *In Vitro* Bovine Ruminal Microbial Fermentations

**DOI:** 10.1155/2013/398320

**Published:** 2013-01-17

**Authors:** Heather A. Duoss-Jennings, Ty B. Schmidt, Todd R. Callaway, Jeffery A. Carroll, James M. Martin, Sara A. Shields-Menard, Paul R. Broadway, Janet R. Donaldson

**Affiliations:** ^1^Department of Animal Science, Iowa State University, Ames, IA, USA; ^2^Animal Science Department, University of Nebraska, Lincoln, NE, USA; ^3^Food and Feed Safety Research Unit, ARS, USDA, College Station, TX 79403, USA; ^4^Livestock Issues Research Unit, ARS, USDA, Lubbock, TX 77845, USA; ^5^Department of Animal and Dairy Sciences, Mississippi State University, Mississippi State, MS, USA; ^6^Department of Biological Sciences, Mississippi State University, Mississippi State, MS 39762, USA; ^7^Department of Animal and Food Sciences, Texas Tech University, Lubbock, TX, USA

## Abstract

Citrus byproducts (CBPs) are utilized as a low cost nutritional supplement to the diets of cattle and have been suggested to inhibit the growth of both *Escherichia coli* O157:H7 and *Salmonella*. The objective of this study was to examine the effects *in vitro* that varying concentrations of CBP in the powdered or pelleted variety have on the survival of Shiga-toxin *Escherichia coli* (STEC) serotypes O26:H11, O103:H8, O111:H8, O145:H28, and O157:H7 in bovine ruminal microorganism media. The O26:H11, O111:H8, O145:H28, and O157:H7 serotypes did not exhibit a change in populations in media supplemented with CBP with either variety. The O103:H8 serotype displayed a general trend for an approximate 1log_10_ reduction in 5% powdered CBP and 20% pelleted CBP over 6 h. There was a trend for reductions in populations of a variant form of O157:H7 mutated in the *stx*1 and *stx*2 genes in higher concentrations of CBP. These results suggest that variations exist in the survival of these serotypes of STEC within mixed ruminal microorganism fluid media when supplemented with CBP. Further research is needed to determine why CBPs affect STEC serotypes differently.

## 1. Introduction

Shiga-toxin producing *Escherichia coli* (STEC) is capable of naturally colonizing within the gastrointestinal tract of cattle without causing illness [1]. Human consumption of products contaminated with STEC can cause the severe illnesses hemorrhagic colitis and hemorrhagic uremic syndrome [2, 3]. The most notorious STEC within the meat industry has been *E. coli* O157:H7. Due to increased surveillance and pre- and post-harvest intervention, the occurrence of O157:H7 infections in the United States has been reduced to ≤1 case per 100,000 people. However, there now appears to be an increase in the occurrence of foodborne outbreaks due to non-O157 STEC serotypes. According to the Center for Disease Control (CDC) an estimated 265,000 cases of STEC infections were reported a year; of these, approximately 67% are attributed to non-O157 STEC [4]. With increased concerns related to the prevalence of non-O157 outbreaks, the United States Department of Agriculture Food Safety and Inspection Service (USDA-FSIS) has recently labeled the non-O157 STEC serogroups O26, O45, O103, O111, O121, and O145 as adulterants in fresh nonintact beef products [5]. 

The production of citrus for various food and nonfood products generates byproducts, such as the pulp and peel from citrus fruit. These citrus byproducts (CBPs) have been utilized by dairy and beef cattle producers in some regions of the United States as an inexpensive nutritionally dense feed source [6]. The incorporation of CBP into diets for cattle may also aid in the reduction of foodborne pathogens due to antimicrobial aspects of the byproducts. Citrus products and by-products contain essential oils that possess antimicrobial activities that can damage the cell wall of gram-negative bacteria [7]. The change in the fluidity of the membranes due to the permeabilization allows essential oils to coagulate in the cytoplasm [8], depleting ATP [9] and resulting in lysis of the cell [9, 10]. The rumen and intestinal gram-negative microbial populations of cattle can be altered due to this antimicrobial activity within cattle [11]. Since CBPs contain antimicrobial properties and are readily available at low costs within citrus-producing areas and has nutritional value, it is being investigated as a potential preharvest pathogen intervention strategy to reduce STEC concentrations within the gastrointestinal tract of cattle. Therefore, the objective of this study was to examine the effects that powdered and pelleted citrus by-products have on growth of the STEC serotypes O26:H11, O103:H8, O111:H8, O145:H28, O157: H7, and O157:H7 Δ*stx*1*stx*2 in bovine ruminal microorganism media.

## 2. Materials and Methods

### 2.1. Ruminal Fluid Collection and Medium Preparation

Ruminal contents (1 L) were collected from the rumen ventral sac of a 544 kg cannulated steer at the Henry Leveck Animal Research Center at Mississippi State University. Rumen particles were separated from the ruminal fluid by passing contents through nylon paint strainers as previously described by others [12]. After separation, rumen fluid was incubated for 30 min at 37°C, to allow the fluid to separate into three distinct layers. The middle layer of the rumen fluid was extracted and utilized for the mixed ruminal microorganism medium. The base medium utilized for the mixed ruminal microorganism fluid contained (per liter): 6.0 g KH_2_PO_4_, 6.0 g KH_2_PO_4_, 12.0 g (NH_4_)_2_SO_4_, 12.0 g NaCl, 2.5 g MgSO_4_·7H_2_O, 1.6 g CaCl_2_·2H_2_O, 0.04 g cysteine HCl; base medium was sterilized by autoclaving [13]. To the base medium, 33.0 mL of an 8% solution of Na_2_CO_3_ and 333 mL of ruminal fluid were added and homogenized by mixing. The pH was adjusted to 6.5 with a 1 M NaOH solution and bubbled with CO_2_. The fully prepared medium was incubated in an orbital shaker incubator at 140 rpm at 37°C for 12 h.

### 2.2. Bacterial Serotypes and Cultivation Conditions

 Six strains of *E. coli* included in this study were purchased from the American Type Culture Collection (ATCC): O157:H7 (ATCC 43895), O103:H8 (ATCC 23982), O145:H28 (BAA-2129), O26:H11 (BAA-1653), O111:H8 (BAA-179), and O157:H7 Δ*stx*1*stx*2 (ATCC 43888). All *E. coli *strains were routinely cultured in Luria-Bertani medium (LB; Difco Co.; Corpus Christi, TX, USA) at 37°C. All strains were transformed with the bioluminescent pXEN-13 plasmid (Caliper Life Sciences; Hopkinton, MA, USA) as a means for identification after incubation. For transformation with the pXEN-13 plasmid, all strains were made competent by washing midlog cultures four times with ice cold 10% glycerol. Competent cells were then transformed with pXEN-13 by electroporation and cultured in LB supplemented with 100 *μ*g/mL of ampicillin (AMP) using standard techniques [14].

### 2.3. Citrus Byproduct Trial

Isolates from fresh streaks of each strain transformed with pXEN-13 were used to inoculate a 5 mL starter culture of LB broth supplemented with AMP for 16 h at 37°C. Cultures were then diluted 1 : 100 in 5 mL LB broth and allowed to grow to mid-log phase (OD_600_ = 0.05), after which cultures were pelleted, residual medium was removed, and cells were resuspended in 5 mL of mixed ruminal microorganism fluid medium supplemented with 0, 5, 10, or 20% CBP (w/v). Cultures were incubated at 37°C in a shaker incubator for 6 h. Aliquots (0.1 mL) were removed at 0, 2, 4, and 6 h after incubation in ruminal medium, serially diluted in 1X phosphate buffered saline (PBS), plated onto LB agar supplemented with AMP, and incubated overnight at 37°C. The pH values were measured from each strain at each time interval at the various CBP concentrations recorded.

### 2.4. Statistical Analysis

Data were analyzed as a completely randomized design with repeated measures using PROC MIXED in SAS (SAS Inst. Inc., Version 9.2; Cary, NC). Experimental unit was defined as tube, and significance was declared at *P* < 0.05. Pair-wise differences among least squares means at various sample times were evaluated with the PDIFF statement.

## 3. Results and Discussion

A study recently conducted by our group using the serotypes analyzed in this study (O26:H11, O103:H8, O111:H8, O145:H28, O157:H7, and O157:H7 Δ*stx*1*stx*2) suggested that all serotypes were capable of growing within mixed ruminal microorganism fluid media; however decreased populations of serotypes O103:H8 and O145:H28 were observed after 24 h in comparison to O157:H7 [15]. These data suggest the possibility that not all non-O157 serotypes function similarly within cattle. To expand upon this previous study, the effect of CBP on non-O157 STEC was analyzed *in vitro*. 

The growth of the various non-O157 STEC (log_10_ CFU/mL) was analyzed within mixed ruminal microorganism fluid medium supplemented with powdered CBP (Table 1). The O26:H11 and O145:H28 serotypes grew similar (*P* < 0.11) within the powdered CBP, with an exception of a decrease in O26:H11 populations (*P* < 0.006) at 4 h in the presence of 20% powdered CBP. The O103:H8 serotype exhibited approximately a 1log⁡_10_ reduction in populations over the 6 h study in the presence of 5% powdered CBP. The O157:H7 Δ*stx*1*stx*2 and O157:H7 serotypes had decreased populations (*P* < 0.04 and *P* < 0.05, resp.) in comparison to the other serotypes at 0 h. Although both O157 serotypes tended to grow similarly (*P* < 0.06) for 4 h, there was a difference observed at 6 h when O157:H7 Δ*stx*1*stx*2 had significantly lower populations (*P* < 0.03) in comparison to O157:H7. When supplemented with 10% powdered CBP, the O157:H7 Δ*stx*1*stx*2 strains displayed approximately a 1.5log⁡_10_ reduction in populations, and at 20% powdered CBP there was approximately a 5log⁡_10_ reduction in populations over 6 h.

Variations were also observed in certain STEC serotypes when grown in the presence of mixed ruminal microorganism fluid medium supplemented with pelleted CBP (Table 2). The pelleted CBP tended to have no change (*P* < 0.07) in populations of O145:H28 from 0 h to 6 h. While O157:H7 had decreased populations (*P* < 0.02) at 0 h, there were no differences in populations observed between the O103:H8 and O157:H7 (*P* < 0.11) the remainder of the study. Populations of O103:H8 were decreased (*P* < 0.02) at 0 h, while populations tended to be similar (*P* < 0.06) to O111:H8 during the 6 h analyzed in this study. When O103:H8 was cultured in the mixed ruminal microorganism medium supplemented with 20% pelleted CBP, there was a general trend for approximately a 1log⁡_10_ reduction observed over the 6 h study. The O26:H11 serotype populations decreased (*P* < 0.05) throughout the study. *Escherichia coli *O157:H7 Δ*stx*1*stx*2 exhibited the lowest populations (*P* < 0.05) throughout the study. 

The O157:H7 Δ*stx*1*stx*2 had reduced populations in mixed rumen microorganism fluid medium supplemented with powdered CBP, while O103:H8 had decreased populations within both varieties of CBP. These results are in accordance with previous studies that have suggested a decrease in O157:H7 populations using other varieties of CBP. A study conducted by Callaway et al. supplemented mixed ruminal microorganism fluid media with 0%, 0.5%, 1%, and 2% dried orange pulp and *E. coli* O157:H7 populations decreased according to increasing concentrations [6]. Reductions in *E. coli *O157:H7 populations have also been observed when sheep rations were supplemented with 5% or 10% orange peel [16].

The CBP was added to the mixed ruminal microorganism fluid media 2 h before the serotypes were added to the mixture (0 h). Although a decrease in O103:H8 and O157:H7 Δ*stx*1*stx*2 populations was observed, other STEC populations were not affected. In the presence of CBP in either pelleted or powdered form, the pH for all strains was reduced from ~5.0 to ~4.0, while in control groups the pH increased from ~6.6 to 7.3. Therefore, the reductions in populations must be attributed to the CBP, as the pH variations were consistent between all strains. Given that this study was only conducted for 6 h, the effects of CBP within the mixed ruminal microorganism fluid media and STEC serotypes may not had been fully observed within the short time frame. This is potentially due to the diffusion properties of CBP across the cell envelope of *E. coli*. Others studies indicate that CBPs decrease *E. coli *O157:H7 populations from 24 h to 72 h [6, 16]. This study was only conducted for 6 h; an increased duration of the study could have been more beneficial to observe the effects of CBP on the various serotypes.

The essential oils within the CBP can permeabilize the bacterial cells walls and cytoplasm, leading to bacterial lysis, thus shifting the rumen environment leading to an increase in short-chain fatty acids while decreasing the pH. The acidic environment creates less favorable conditions for microbial populations to survive and replicate within, thus decreasing the possibility of *E. coli* O157:H7 populations. Although our research has reported a decrease in pH values with increasing CBP concentrations and an observed decrease in O103:H8 and O157:H7 Δ*stx*1*stx*2 populations, this same trend was not observed within other STEC serotypes. Further research is needed to determine how the various STEC serotypes affect *E. coli* populations within mixed ruminal microorganism fluid media when supplemented with CBP.

## Figures and Tables

**Table 1:** Least squares means for growth of  STEC O26:H11, O103:H8, O111:H8, O145:H28, O157:H7, and O157:H7 Δ*stx*1*stx*2 within bovine mixed rumen microorganisms fluid medium, supplemented with 0%, 5%, 10%, and 20% powdered citrus by-products (CBPs).

CBP	Time (h)	*E. coli* serotype (Log_10_ CFU/mL)					
		O26:H11	O103:H8	O111:H8	O145:H28	O157:H7	O157:H7 Δ*stx*1*stx*2
	0 h	7.55	7.11	6.93	7.55	6.03^v^	6.46
	2 h	7.68	7.05	6.70	7.58	6.91^w^	7.00
0%	4 h	7.60	6.81	6.92	8.06	6.91^w^	7.19
	6 h	7.73	6.65	7.07	7.83	7.24^w^	6.98
	Δ^a^	0.18	−0.046	0.14	0.28	1.21	0.52

	0 h	7.40	7.34^y^	7.31^v^	7.57	6.73	6.63
	2 h	7.47	6.86^yz^	7.06^v^	7.64	6.65	6.41
5%	4 h	7.43	6.95^yz^	5.85^w^	7.80	6.77	7.09
	6 h	7.59	6.55^z^	7.22^v^	7.80	7.02	6.74
	Δ	0.23	−0.59	0.61	−0.1	0.15	−2.63

	0 h	7.43	7.28	6.64	7.73	6.83	6.82^vw^
	2 h	7.43	6.90	7.33	7.56	7.22	6.18^w^
10%	4 h	7.36	6.99	7.18	7.64	6.96	7.13^v^
	6 h	7.66	6.69	7.25	7.63	6.98	4.19^x^
	Δ	0.07	−0.47	−0.32	0.06	−0.03	−5.04

	0 h	7.12^v^	7.13	7.02	7.60	7.03	7.03^v^
20%	2 h	7.54^v^	7.33	6.78	7.52	6.94	6.53^v^
	4 h	5.96^w^	7.07	6.65	7.44	7.30	7.34^v^
	6 h	7.19^v^	6.66	6.70	7.66	7.00	1.99^w^

^a^Change in concentration between 0 h and 6 h.

^v,w,x^Lsmeans within a column, within a treatment, without a common subscript are different if *P* ≤ 0.05.

^y,z^Lsmeans within a column, within a treatment, without a common subscript tend to be different if *P* < 0.09.

**Table 2:** Least squares means for growth of STEC O26:H11, O103:H8, O111:H8, O145:H28, O157:H7, and O157:H7 Δ*stx*1*stx*2 within bovine mixed rumen microorganisms fluid medium, supplemented with 0%, 5%, 10%, and 20% pelleted citrus by-products (CBPs).

CBP	Time (h)	*E. coli* serotype (Log_10_ CFU/mL)					
		O26:H11	O103:H8	O111:H8	O145:H28	O157:H7	O157:H7 Δ*stx*1*stx*2
	0 h	9.27^x^	7.01^x^	8.47^x^	9.61	8.48	6.46
	2 h	8.08^y^	7.01^x^	9.01^xy^	9.48	8.24	6.27
0%	4 h	9.45^x^	8.64^y^	8.43^x^	9.67	8.79	6.26
	6 h	9.64^x^	8.77^y^	9.48^y^	9.50	9.01	6.51
	Δ^a^	0.37	1.76	1.01	−0.11	0.53	0.05

	0 h	9.27^xy^	8.21	8.40^xy^	9.51	9.23^x^	6.07
	2 h	8.71^x^	8.26	8.93^x^	9.60	7.94^y^	5.92
5%	4 h	7.29^z^	7.93	7.98^y^	9.59	7.96^y^	6.23
	6 h	8.71^y^	8.28	8.77^x^	9.69	8.93^x^	5.97
	Δ	−0.56	0.07	0.37	0.18	−0.3	−0.1

	0 h	9.07^xz^	8.76	8.76^xy^	8.98	8.73^xy^	6.83
	2 h	8.43^xy^	8.50	9.03^xy^	9.01	8.73^xy^	6.94
10%	4 h	7.90^y^	8.37	8.66^x^	9.55	8.40^xy^	6.64
	6 h	8.59^z^	8.07	9.73^y^	9.71	9.20^y^	6.68
	Δ	−0.48	−0.69	0.97	0.73	0.47	−0.15

	0 h	9.47^x^	8.52^x^	9.03	9.29^x^	8.00^x^	6.78
	2 h	8.98^x^	8.51^x^	8.92	9.79^xy^	8.00^y^	6.76
20%	4 h	7.69^y^	8.57^x^	9.09	8.95^y^	8.71^xy^	6.76
	6 h	9.54^x^	7.43^y^	9.46	9.48^xy^	9.05^y^	7.13
	Δ	−0.07	−1.09	−0.43	0.19	1.05	0.35

^a^Change in concentration between 0 h and 6 h.

^x,y,z^Lsmeans within a column, within a treatment, without a common subscript are different if *P* ≤ 0.05.
